# Alpha Lipoic Acid Modulated High Glucose-Induced Rat Mesangial Cell Dysfunction via mTOR/p70S6K/4E-BP1 Pathway

**DOI:** 10.1155/2014/658589

**Published:** 2014-10-30

**Authors:** Chuan Lv, Can Wu, Yue-hong Zhou, Ying Shao, Guan Wang, Qiu-yue Wang

**Affiliations:** ^1^Division of Endocrinology, First Affiliated Hospital, China Medical University, Shenyang, Liaoning 110001, China; ^2^Division of Endocrinology, Shenyang No. 8 Hospital, Shenyang, Liaoning 110024, China; ^3^Clinical Medicine of Seven-Year Education, China Medical University, Shenyang, Liaoning 110001, China

## Abstract

The aim of this study was to investigate whether alpha lipoic acid (LA) regulates high glucose-induced mesangial cell proliferation and extracellular matrix production via mTOR/p70S6K/4E-BP1 signaling. The effect of LA on high glucose-induced cell proliferation, fibronectin (FN), and collagen type I (collagen-I) expression and its mechanisms were examined in cultured rat mesangial cells by methylthiazol tetrazolium (MTT) assay, flow cytometry, ELISA assay, and western blot, respectively. LA at a relatively low concentration (0.25 mmol/L) acted as a growth factor in rat mesangial cells, promoted entry of cell cycle into S phase, extracellular matrix formation, and phosphorylated AKT, mTOR, p70S6K, and 4E-BP1. These effects disappeared when AKT expression was downregulated with PI3K/AKT inhibitor LY294002. Conversely, LA at a higher concentration (1.0 mmol/L) inhibited high glucose-induced rat mesangial cell proliferation, entry of cell cycle into S phase, and extracellular matrix exertion, as well as phosphorylation of mTOR, p70S6K, and 4E-BP1 but enhanced the activity of AMPK. However, these effects disappeared when AMPK activity was inhibited with CaMKK inhibitor STO-609. These results suggest that LA dose-dependently regulates mesangial cell proliferation and matrix protein secretion by mTOR/p70S6K/4E-BP1 signaling pathway under high glucose conditions.

## 1. Introduction

Diabetic nephropathy (DN) is one of the most serious microvascular complications of diabetes and the leading cause of end-stage renal disease [1]. The prevalence of DN is growing dramatically despite considerable attention from the public [2, 3]. Mesangial cell (MC) proliferation and excessive mesangial extracellular matrix (ECM) have been identified as contributing factors to the initial pathophysiologic mechanisms involved in glomerulosclerosis, which is typical of DN [4]. Thus, developing effective approaches to inhibit mesangial cell proliferation and ECM accumulation is important for the prevention of glomerulosclerosis in diabetes.

LA, a powerful antioxidant that plays an important role in regulating glucose and lipid metabolism, as well as attenuating deposition of mesangial matrix [5], has gained considerable attention because of its use in managing diabetic complications. As a naturally occurring short-chain fatty acid, LA can activate AMPK in the hypothalamus and peripheral tissues [6–10]. Ca^2+^/calmodulin-dependent protein kinase II (CaMKII) acts as the upstream of AMP-activated protein kinase in mammalian cells [10]. LA has also been shown to activate AMPK and suppress mTOR/p70s6K signaling in rat skeletal muscle by increasing CaMKII, thereby improving insulin resistance [11]. Several recent studies have found that LA also activates the AKT pathway through direct binding to the tyrosine kinase domain of insulin receptors [12–14]. Administration of LA has been shown to prevent ischemia and reperfusion-induced cerebral endothelial cell injury by upregulating the phosphorylation of Akt, mTOR, p70s6K, and 4E-BP1 [15]. These findings suggest that LA might regulate mTOR signaling by activating AKT and AMPK.

mTOR, the mammalian target of rapamycin, is a serine/threonine kinase that forms a part of two functionally distinct protein complexes, mTORC1 and mTORC2. mTORC1 consists of four subunits: mTOR, mLST8, PRAS40, and the raptor, each playing an important role in regulating cell growth and proliferation by directly phosphorylating two regulators of protein translation, p70-S6 kinase (p70S6K) and 4E binding protein 1 (4E-BP1) [16]. The AKT pathway activates mTORC1 by two mechanisms: (1) AKT activation phosphorylates TSC2 and inhibits its GAP activity, thus stimulating mTOR and mTORC1, and (2) AKT causes the phosphorylation of PRAS40 to alleviate its mTORC1 inhibitory effect [17]. When active, AMPK inhibits mTORC1 by regulating phosphorylation of TSC2 and raptor [18]. In diabetes, hyperglycemia increases mTOR activity by the combined effects of AKT activation and AMPK inhibition [19]. Activation of mTOR results in renal changes to DN, including glomerular hypertrophy, deposition of mesangial matrix, and glomerular basement membrane thickening [20].

The current study examined the effects of LA on cell proliferation and ECM secretion in MCs. It also sought to determine whether the effect was mediated by the mTOR/p70S6K/4E-BP1 signaling pathway.

## 2. Materials and Methods

### 2.1. Cell Culture

HBZY-1 cells (MCs), a rat glomerular mesangial cell line obtained from the China Center for Type Culture Collection, Wuhan, China, were thawed and cultured in GIBCO MEM (Life Technologies Inc., Grand Island, NY, USA) containing 10% fetal bovine serum (Abgent, LGC Biotecnologia Ltda, Sao Paulo, Brazil), 100 units/mL penicillin, and 100 *μ*g/mL streptomycin in a 5% CO_2_ incubator at 37°C. The medium was changed every 2 days. Passages 10–15 of the cells were used in this study. After preincubation overnight in MEM without fetal bovine serum, cells were used for subsequent experiments. Cells grown in normal glucose control were treated with 5.5 mmol/L D-glucose. In the osmotic control group, MCs were incubated in normal glucose medium supplemented with 25 mmol/L mannitol. Cells were incubated with medium supplemented with 30 mmol/L D-glucose in high glucose group.

### 2.2. Reagents

LA was purchased from Sigma-Aldrich (St. Louis, MO, USA). LA was added to the incubation medium after 1 h stimulation of high glucose (30 mmol/L D-glucose) to produce final concentrations ranging from 0 to 1 mM in the medium. PI3K/AKT specific inhibitor LY294002 was purchased from Cell Signaling Technology, Inc. (Beverly, MA, USA). The CaMKK specific inhibitor STO-609 acetic acid was purchased from Sigma-Aldrich. Cells on culture were treated with LY294002 or STO-609 for 30 min prior to LA administration.

### 2.3. Cell Proliferation Assay

The MTT assay was used to detect cell proliferation. We cultured MCs in 96-well plates (5 × 10^3^cells/well). After 12, 24, and 48 h incubation with different compounds as described above, 20 *μ*L MTT (5 mg/mL, Invitrogen, USA) was added to each well. Cells were then cultured for an additional 4 h and subsequently lysed using dimethylsulfoxide (100 *μ*L/well; Sigma, USA). When the formazan crystals completely dissolved, the optical density (OD) was measured at 570 nm using a Biotek Synergy microplate reader (Biotek, Winooski, VT, USA). The arithmetic mean OD of six wells for each group was calculated.

### 2.4. Flow Cytometry

Cell-cycle analysis was performed usingflow cytometry as described previously [21] with some modification. After treatment with indicated compounds, cells were harvested 24 h after incubation, washed twice with cold phosphate buffer saline (PBS) buffer, and then fixed with 70% alcohol for 12 h at 4°C. After washing, cells were treated with RNase (50 *μ*g/mL) at 37°C for 30 min. Cells were stained with propidium iodide (PI, 50 *μ*g/mL) at 4°C for 30 min in the dark before being analyzed with a BD FACSCalibur flow cytometer (BD Biosciences, Franklin Lakes, New Jersey, USA). 1 × 10^6^ cells were detected for each sample and the cell cycle was analyzed using BD CellQuest software (BD Biosciences, USA).

### 2.5. ELISA Assay

Cell culture supernatants from different treatment groups were harvested and centrifuged at 2000 g for 20 min. After centrifugation, the supernatants were then assayed for FN and collagen-I secretions using the ELISA kits (Boster Biological Engineering Co., Wuhan, China) [22]. Each sample was analyzed in triplicate. The absorbance was measured at 450 nm wavelength with a Biotek Synergy microplate reader (Biotek, Winooski, VT, USA).

### 2.6. Quantitative Real-Time PCR

Cell total RNA was extracted using Trizol RNA isolation reagent (Takara Bio Inc, Shiga, Japan). One *μ*g of total RNA was reverse-transcripted into cDNA according to manufacturer instructions using a Takara RT kit. Real-time PCR was performed using a SYBR Premix Ex Taq II reagent kit (Takakra). The primer sets used were

 5′-TGAAGATCGGCCACTACATCCT-3′,
 5′-CACAGCAACTTTATGTCCAGTCAAC-3′ for AMPK*α*,
 5′-TGACAGGATGCAGAAGGAGATTAC-3′,
 5′-GAGCCACCAATCCACACAGA-3′ for beta-actin.

The reaction volume was 25 *μ*L; 2 *μ*L cDNA was used as a template. PCR amplification was performed under the following conditions: initial denaturation at 95°C for 30 s followed by 40 cycles of denaturation at 95°C for 5 s; annealing at 60°C for 30 s; and extension at 72°C for 30 s. Fluorescence was detected using the Takakra Thermal Cycler Dice Real Time System. Experiments were repeated in triplicate. Data analysis based on measurements of the threshold cycle was performed using the 2^−ΔΔCt^ method [23].

### 2.7. Protein Extraction and Western Blotting

Western blot was performed as described previously [15, 24]. In brief, after treatment with different compounds, cells were harvested and lysed in RIPA lysis buffer containing PMSF protease inhibitors. Protein concentrations were measured by a BCA assay (Beyotime Institute of Biotechnology, Shanghai, China) using BSA as a standard. Samples were boiled at 100°C for 10 min in 5× sample buffer. Equal amounts of protein (60 *μ*g per sample) were separated by 4%–15% SDS-PAGE and then transferred onto a PVDF membrane. After blocking with 5% BSA in Tris buffered saline Tween 20 (TBS-T, pH 7.6; Sigma-Aldrich, Shanghai, China) for 1.5 h at room temperature, membranes were probed with primary antibodies overnight at 4°C. The manufacturer and catalog numbers of all of the primary antibodies used are listed in Table 1. After extensive washing, the membranes were incubated with anti-rabbit IgG secondary antibody (1 : 5000). The membranes were then reacted with ECL-Plus chemiluminescent detection HRP reagents (Beyotime). Immunoreactive bands were visualized using a MicroChemi 4.2 Bioimaging system (Jerusalem, Israel), and the densitometry of phosphorylated protein was normalized to its corresponding total protein for assessing protein activation.

### 2.8. Statistical Analyses

Data obtained from at least three independent experiments are expressed as mean ± SD. Statistical comparisons between multiple groups were performed by one-way ANOVA, and the Holm-Bonferroni method was applied to control for multiple testing (GraphPad Prism 5.0; GraphPad Software, La Jolla, CA, US). In all cases, *P* < 0.05 was considered to represent statistical significance.

## 3. Results

### 3.1. LA Dose-Dependently Regulated High Glucose-Induced Rat Mesangial Cell Proliferation

**Figure 1:**
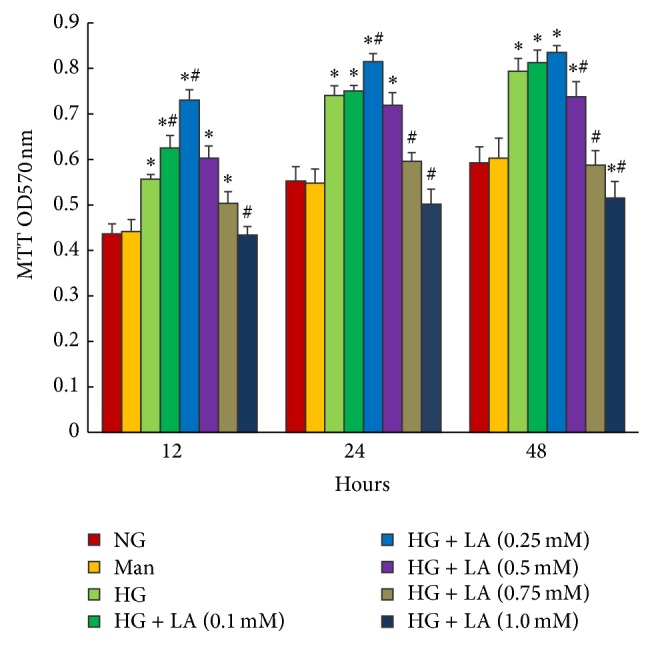
LA dose-dependently regulated high glucose-induced proliferation of rat mesangial cells. Proliferation of mesangial cells was determined by MTT assay. LA accelerated mesangial cell proliferation induced by high glucose for 12, 24, or 48 h, at final concentrations of 0.1, 0.25 mmol/L, respectively, while cell proliferation caused by high glucose was inhibited as the dose of LA increased to >0.5 mmol/L. Values are given as mean ± SD (*n* = 6) and *P* < 0.05 was considered statistically significant. Note: ^*^ *P* < 0.05 versus NG; ^#^ *P* < 0.05 versus HG. Abbreviations: NG, normal glucose (5 mM); Man (5 mM glucose + 2 5 mM mannitol); HG, high glucose (30 mM).

MTT assay was performed to assess the proliferation of MCs treated with LA (0.1–1 mM). As shown in Figure 1, high glucose induced cell proliferation. It was interestingly noted that administration with 0.1 and 0.25 mmol/L for 12, 24, or 48 h significantly promoted high glucose-induced MC proliferation, with a maximum increment in 0.25 mmol/L for 24 h. However, LA arrested cell proliferation as the dosage increased above 0.5 mmol/L, with a maximum inhibition at 1.0 mM.

### 3.2. LA Concentration-Dependently Regulated Cell-Cycle Progression in High Glucose-Treated Rat Mesangial Cells

**Figure 2:**
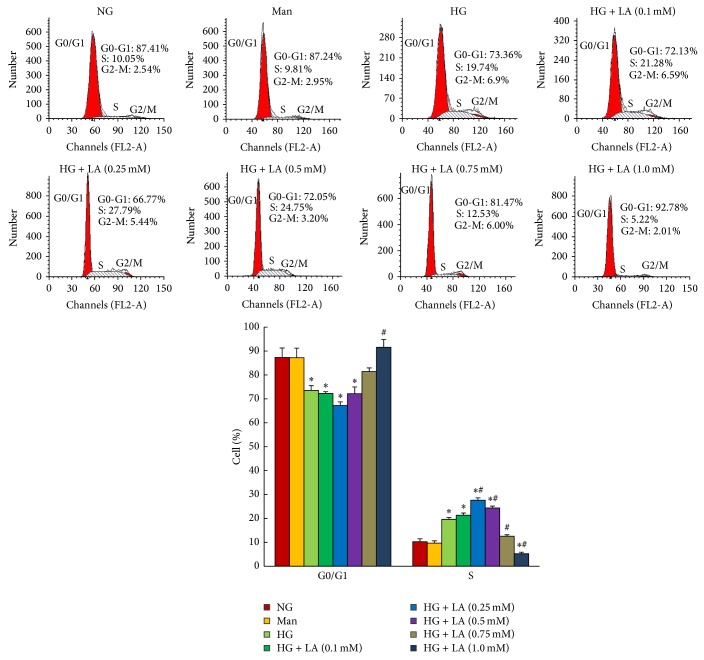
LA concentration-dependently modulated mesangial cell-cycle progression stimulated by high glucose. MCs were preincubated in medium with high glucose for 1 h and then stimulated with various concentrations of LA for 24 h, respectively. LA with final concentrations of 0.1 and 0.25 mM promoted cell-cycle progression induced by high glucose, with a maximum promotion at 0.25 mM, while cell cycle was inhibited as the dose of LA increased above 0.5 mM, with a maximum inhibition at 1 mM. Cells were analyzed by flow cytometry after PI staining and the relative percentage of cells in different cell-cycle phases is reported, while the percentage of apoptotic events was ignored. Data in the bar graphs represent the mean ± SD (*n* = 3). *P* < 0.05 was considered statistically significant. Note: ^*^ *P* < 0.05 versus NG; ^#^ *P* < 0.05 versus HG. Abbreviations: NG, normal glucose (5 mM); Man (5 mM glucose + 25 mM mannitol); HG, high glucose (30 mM).

To further examine the effects of LA on cell-cycle progression, we performed flow cytometry analysis. As shown in Figure 2, compared with normal glucose control, high glucose induced a 16% (*P* < 0.001) decrease in cell proportion in G0/G1 phase and a 96.4% (*P* < 0.001) increase in S phase, indicating that high glucose promoted cell-cycle progression. Compared to the high glucose-treated group, 0.25 mmol/L caused a 40.8% increase in cells in S phase (*P* < 0.001) and a 9% decrease in cells in G0/G1 phase (*P* > 0.05). In contrast, 1 mmol/L induced a 26.5% (*P* < 0.001) increase of cells in G0/G1 phase and a 73.6% decrease in S phase. These results suggest that LA exhibited a twofold effect on glucose-induced cell-cycle progression based on different dosages.

### 3.3. Determination of the Dose-Dependent Effect of LA on the Secretion of FN and Collagen-I in Cell Culture Supernatants

**Figure 3:**
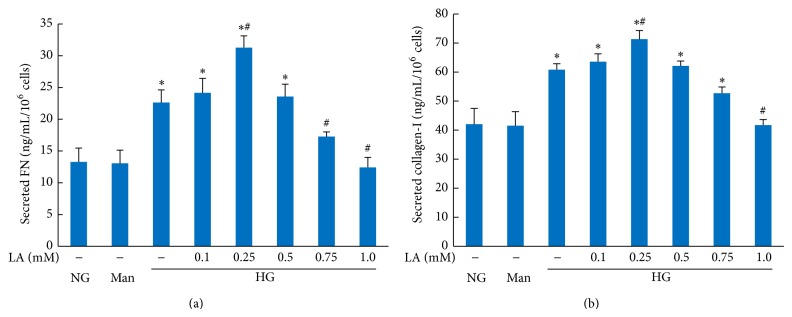
Effect of LA on the secretions of FN and collagen-I in high glucose-treated mesangial cells. Supernatants were assayed by ELISA after treatment with different concentration of LA for 24 hours. Values are given as mean ± SD from 3 independent experiments in quadruplicate and *P* < 0.05 is considered statistically significant. Note: ^*^ *P* < 0.05 versus NG; ^#^ *P* < 0.05 versus HG. Abbreviations: NG, normal glucose (5 mM); Man (5 mM glucose + 25 mM mannitol); HG, high glucose (30 mM).

The expression of FN and collagen-I in cell culture supernatants is summarized in Figure 3. The secretion of FN and collagen-I was higher from MCs in the high glucose group than in the control group, and expression was further upregulated after treatment with 0.1 and 0.25 mmol/L LA. However, higher doses of LA (0.75 and 1.0 mmol/L) reduced the secretion of both FN and collagen-I.

### 3.4. LA Concentration-Dependently Modulated the Expression of AMPK, AKT, mTOR, p70S6K, and 4E-BP1 in High Glucose-Cultured Mesangial Cells

**Figure 4:**
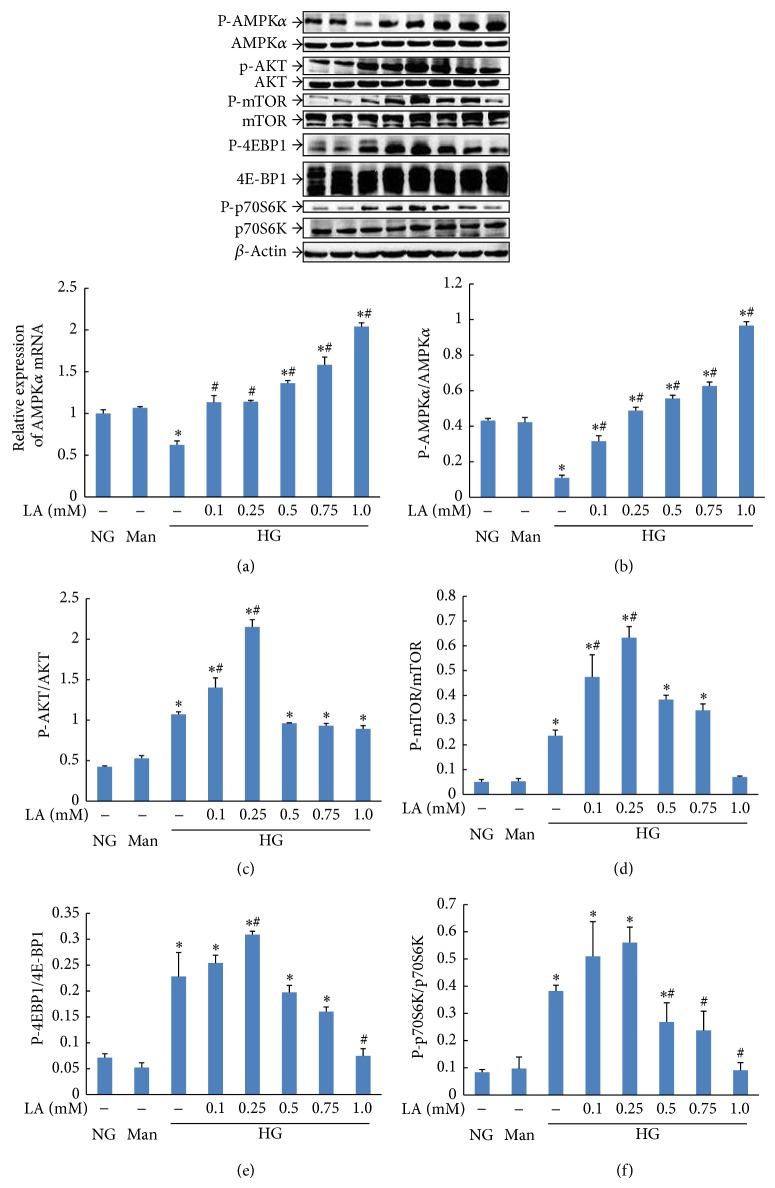
Concentration-dependent LA regulated expressions of AMPK, AKT, mTOR, 4E-BP1, and p70S6K in high glucose-treated mesangial cells. MCs were preincubated for 1 h with high glucose (30 mmol/L) and then stimulated with various concentrations (0.1–1.0 mmol/L) of LA for 24 h, respectively. The relative quantitation of AMPK*α* mRNA was expressed as the ratio of AMPK*α*/*β*-actin (a), which in normal glucose cells was set to 1. Phosphorylation of AMPK*α* (b), AKT (c), mTOR (d), 4E-BP1 (e), and p70S6K (f) was determined by immunoblot analysis. *β*-Actin was used as a control to ensure equal protein loading. Representative blots (top) and densitometric analyses (bottom) are shown. Values are given as mean ± SD from three independent experiments; *P* < 0.05 was considered statistically significant. Note: ^*^ *P* < 0.05 versus NG; ^#^ *P* < 0.05 versus HG. Abbreviations: NG, normal glucose (5 mM); Man (5 mM glucose + 25 mM mannitol); HG, high glucose (30 mM).

To investigate whether AMPK/mTOR and AKT/mTOR pathways were involved in the observed LA-induced cell proliferation regulative effect, we examined expression of AMPK, AKT, mTOR, 4E-BP1, and p70S6K by real-time PCR and/or western blotting in rat mesangial cells. As shown in Figure 4, high glucose (30 mmol/L for 24 h) resulted in decreased levels of AMPK*α* mRNA expression (Figure 4(a)) (*P* < 0.05) and AMPK phosphorylation (Figure 4(b)) (*P* < 0.001) in MCs compared with those of control cells. Incubation of LA in various concentrations (0.1–1.0 mM) for 24 h resulted in a dose-dependent increase in AMPK*α* mRNA expression (*P* < 0.05) and phosphorylation of AMPK-*α* at Thr172 (*P* < 0.05) compared with that in the high glucose group. Meanwhile, we observed that high glucose (30 mmol/L for 24 h) increased phosphorylation of AKT (Figure 4(c)) and that treatment with 0.1 and 0.25 mM LA enhanced this effect. Treatment with 0.25 mM LA led to the highest activity of AKT in MCs. However, AKT activity in 0.5, 0.75, and 1.0 mM LA-treated MCs showed no difference from that of the high glucose group. In addition, MCs in the high glucose group showed higher levels of mTOR (Figure 4(d)), 4E-BP1 (Figure 4(e)), and p70S6K (Figure 4(f)) phosphorylation than those of the normal glucose group (*P* < 0.05). Treatment with 0.1 and 0.25 mM LA further upregulated levels of mTOR, 4E-BP1, and p70S6K phosphorylation. Of note, administration of 1.0 mM LA completely reversed this situation.

These data suggest that relatively low concentrations of LA (0.25 mM) enhanced mTOR/4E-BP1/p70S6K signaling, while higher doses (1.0 mM) downregulated mTOR signaling. Thus, we selected a dose of 0.25 mM LA and the PI3K/AKT inhibitor LY294002 for subsequent experiments to determine whether LA-induced mTOR activation was mediated by the AKT pathway in MCs. We also selected an mM LA dose of 1.0 mM and the CaMKK inhibitor STO-609 for subsequent experiments to examine whether LA-induced mTOR inhibition was mediated by the AMPK pathway.

### 3.5. The AKT Pathway Is Involved in 0.25 mM LA-Induced Activation of mTOR/p70S6K/4E-BP1 Signaling Pathways: The AMPK Pathway Mediates 1.0 mM LA-Induced mTOR Signaling Inhibition

**Figure 5:**
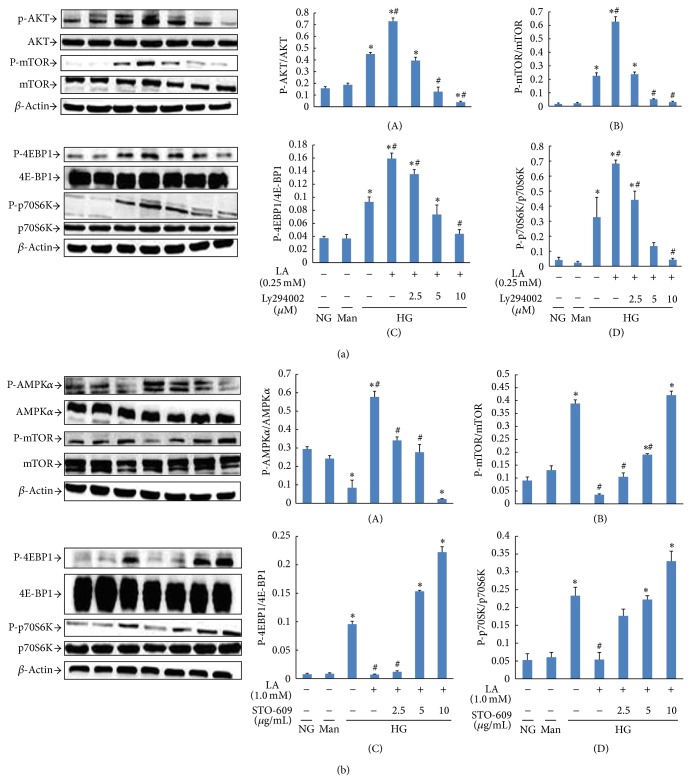
LY294002 prevented the phosphorylation of AKT, mTOR, p70S6K, and 4E-BP1 induced by 0.25 mM LA in high glucose-cultured MCs. Cells were preincubated with high glucose medium for 1 h and then stimulated with different concentrations of LY294002 in the presence of 0.25 mM LA for an additional 24 h. The phosphorylation of AKT ((a) panel (A)), mTOR ((a) panel (B)), 4E-BP1 ((a) panel (C)), and p70S6K ((a) panel (D)) was determined by immunoblot analysis. STO-609 reversed the phosphorylation of AMPK, mTOR, p70S6K, and 4E-BP1 induced by 1.0 mM LA in high glucose-treated MCs. Cells were preincubated with high glucose medium for 1 h and then stimulated with various concentrations of STO-609 in the presence of 1.0 mM LA for 24 h. The phosphorylation of AMPK ((b) panel (A)), mTOR ((b) panel (B)), 4E-BP1 ((b) panel (C)), and p70S6K ((b) panel (D)) in cells was determined by immunoblot analysis. *β*-Actin was used as a control to ensure equal protein loading. Representative blots (left) and densitometric analyses (right) are shown. Values are given as mean ± SD; *P* < 0.05 was considered statistically significant. Note: ^*^ *P* < 0.05 versus NG; ^#^ *P* < 0.05 versus HG. Abbreviations: NG, normal glucose (5 mM); Man (5 mM glucose + 25 mM mannitol); HG, high glucose (30 mM).

To determine whether 0.25 mM LA-induced mTOR/p70S6K/4E-BP1 signaling pathway activation was mediated by the AKT pathway, MCs were pretreated with high glucose (30 mM) for 1 h and then treated with different concentrations of PI3K/AKT inhibitor LY294002 (0, 2.5, 5, or 10 *μ*mol/L) for another 24 h in the presence of 0.25 mM LA. Results showed that levels of AKT, mTOR, p70S6K, and 4E-BP1 phosphorylation in high glucose-cultured MCs were significantly upregulated after 0.25 mM LA stimulation and that LY294002 treatment could dose-dependently reverse this situation (Figure 5(a)).

To examine whether the AMPK pathway was responsible for the mTOR/p70S6K/4E-BP1 signaling inhibition as a result of 1.0 mM LA, MCs were preincubated with high glucose (30 mM) for 1 h and then treated with different concentrations of the CaMKK inhibitor STO-609 (0, 2.5, 5, 10 *μ*g/mL) for another 24 h in the presence of 1.0 mM LA. As shown in Figure 5(b), 1.0 mM LA led to obvious AMPK activation and downregulated levels of mTOR, p70S6K, and 4E-BP1 phosphorylation. STO-609 (above 5 *μ*g/mL) eliminated the effects of 1.0 mM LA on AMPK/mTOR signaling.

These data suggest that LA (0.1, 0.25 mmol/L) could promote high glucose-treated MC proliferation by activating the AKT/mTOR signaling pathway, while higher doses of LA (1.0 mmol/L) could inhibit high glucose-treated MC proliferation by AMPK/mTOR signaling activation.

### 3.6. Inhibition of AKT Suppressed 0.25 mM LA-Induced MC Proliferation and Cell-Cycle Progression: Inhibition of AMPK by STO-609 Abolished the Inhibition of MC Proliferation and Cell-Cycle Progression Caused by 1.0 mM LA

**Figure 6:**
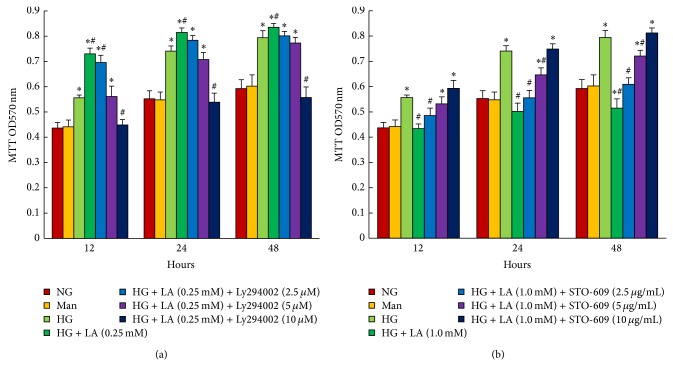
MTT assay for cell proliferation analysis. LY294002 dose-dependently inhibited mesangial cell proliferation induced by 0.25 mM LA. 10 *μ*g/mL STO-609 completely abolished the inhibition of mesangial cell proliferation, which was caused by 1.0 mM LA. Data in the bar graphs represent the mean ± SD (*n* = 6). *P* < 0.05 was considered statistically significant. Note: ^*^ *P* < 0.05 versus NG; ^#^ *P* < 0.05 versus HG. Abbreviations: NG, normal glucose (5 mM); Man (5 mM glucose + 25 mM mannitol); HG, high glucose (30 mM).

We used MTT assay to examine whether the AKT/mTOR signaling pathway was responsible for 0.25 mM LA-induced proliferation of MC cultured in high glucose medium. MCs were pretreated with high glucose (30 mM) for 1 h and then stimulated with various concentrations of LY294004 (0, 2.5, 5, 10 *μ*mol/L) in the presence of 0.25 mM LA for 12, 24, and 48 h, respectively. As shown in Figure 6(a), compared to the high glucose group, 0.25 mM LA treatment promoted MC proliferation. Administration of LY294002 regardless of duration (12, 24, and 48 h) significantly suppressed 0.25 mM LA-induced MC proliferation, with a maximum inhibition in 10 *μ*mol/L (*P* < 0.001). Meanwhile, to determine whether inhibition of MC proliferation resulting from 1.0 mM LA was mediated by AMPK/mTOR signaling, MCs were treated with 30 mM glucose for 1 h, followed by STO-609 (0, 2.5, 5, 10 *μ*g/mL) treatment in the presence of 1.0 mM LA for an additional 12, 24, and 48 h, respectively. Compared to the high glucose group, MC proliferation was significantly arrested when cells were treated with 1.0 mM LA. The inhibition was inhibited by the administration of STO-609 (5 or 10 *μ*g/mL) (*P* < 0.01) (Figure 6(b)).

**Figure 7:**
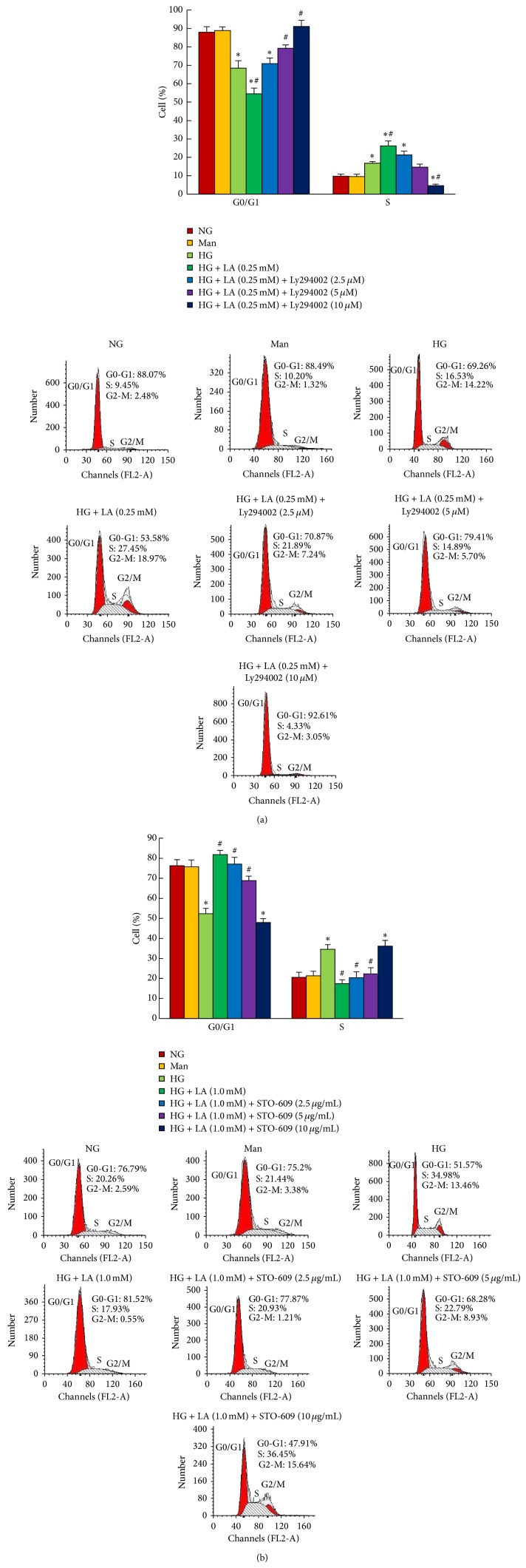
LY294002 inhibited cell-cycle progression induced by 0.25 mM LA in high glucose-cultured MCs. STO-609 prevented the inhibition of high glucose-stimulated mesangial cell-cycle progression caused by 1.0 mM LA. MCs were pretreated with high glucose (30 mM) for 1 h and then incubated with LY294004 (0, 2.5, 5, 10 *μ*mol/L) plus 0.25 mM LA or STO-609 (0, 2.5, 5, 10 *μ*g/mL) plus 1.0 mM LA, respectively, for an additional 24 h. Cells were analyzed by flow cytometry after PI staining. Data in the bar graphs represent the mean ± SD (*n* = 3). *P* < 0.05 was considered statistically significant. Note: ^*^ *P* < 0.05 versus NG; ^#^ *P* < 0.05 versus HG. Abbreviations: NG, normal glucose (5 mM); Man (5 mM glucose + 25 mM mannitol); HG, high glucose (30 mM).

We further examined cell-cycle progression in these cells. AKT inhibitor LY294002 also significantly inhibited G0/G1 cell-cycle progression during 0.25 mmol/L administration in the presence of high glucose, and the number of cells in the S phase decreased, with the maximum inhibition in 10 *μ*mol/L (Figure 7(a)). As shown in Figure 7(b), administration of 1.0 mM LA significantly arrested high glucose-cultured MCs in the G0/G1 phase. As a result, the number of cells at S phase was less than that in high glucose group. This LA-induced cell growth arrest was completely reversed by CaMKK inhibitor STO-609 (10 *μ*g/mL).

### 3.7. Inhibition of AKT Suppressed 0.25 mM LA-Induced FN and Collagen-I Secretion: Inhibition of AMPK by STO-609 Reversed the Inhibition of MC FN and Collagen-I Expression Caused by 1.0 mM LA

**Figure 8:**
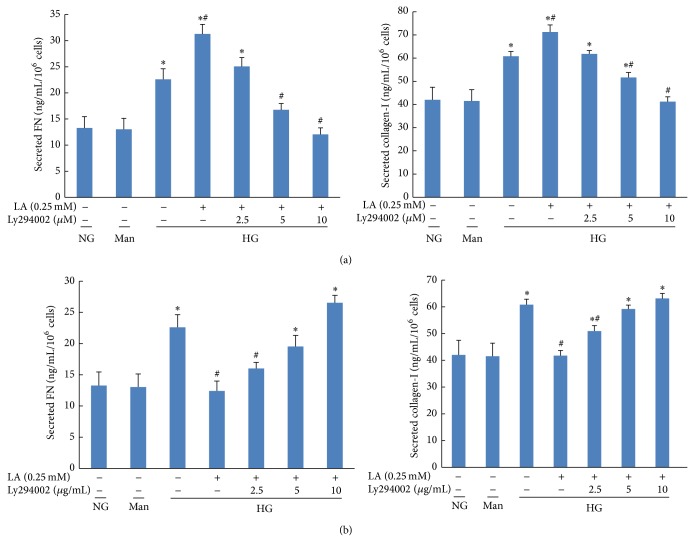
LY294002 prevented the secretions of FN and collagen-I induced by 0.25 mM LA in high glucose-cultured MCs. STO-609 reversed the inhibition of FN and collagen-I secretions induced by 1.0 mM LA in high glucose-treated MCs. (a) Representative of the effects of LY294002 on 0.25 mM LA induced FN and collagen secretion in supernatants. (b) Representative of the effects of STO-609 on 1.0 mM LA induced FN and collagen secretion in supernatants. Values are given as mean ± SD from 3 independent experiments in quadruplicate and *P* < 0.05 is considered statistically significant. Note: ^*^ *P* < 0.05 versus NG; ^#^ *P* < 0.05 versus HG. Abbreviations: NG, normal glucose (5 mM); Man (5 mM glucose + 25 mM mannitol); HG, high glucose (30 mM).

As shown in Figure 8(a), the levels of FN and collagen-I expression increased in high glucose group, and further upregulation was observed in the high glucose group treated with 0.25 mM LA. However, the changes of ECM production caused by 0.25 mM LA were reversed with the addition of LY294002, which inhibited AKT-mTOR signaling. Meanwhile, the effect of 1.0 mM LA on FN and collagen-I secretion in cell culture supernatants were attenuated by STO-609, which reversed the activation of AMPK-mTOR signaling induced by 1.0 mM LA (Figure 8(b)).

## 4. Discussion

LA has been shown to reduce diabetes-induced ROS production, inflammation, and cytokine expression, thereby inhibiting deposition of mesangial matrix proteinsand renal hypertrophy [10, 25]. However, less is known about the effects and molecular mechanisms of LA on high glucose-induced MC proliferation. Recently, Feng et al. observed that diabetes induced mesangial cell proliferation in experimental rats and that 12 weeks of daily administration of LA (35 mg/kg) dissolved in saline via intraperitoneal injection improved diabetes-induced MC proliferation [26]. In agreement with this, the present study showed that high glucose promoted cultured rat renal mesangial cell proliferation. However, for the first time, we observed two effects of LA on high glucose-treated MC cell proliferation: (1) low concentrations (0.1, 0.25 mmol/L) of LA promoted cell proliferation and (2) higher concentrations (1.0 mmol/L) acted as an antiproliferation agent.

For proliferation of MCs, cell cycle responded by flow cytometrywas also assessed. Consistent with previous results [27], the present in vitro study showed that high glucose promoted the cell cycle of MCs from G0/G1 to S phase transition. A very similar trend using MTT flow cytometry assay showed that cells treated with 0.1 and 0.25 mmol/L LA had a significantly lower population at G0/G1 phase, but higher population at S phase, compared with cells cultured under high glucose, while the high glucose-induced G1 to S phase progression of the cell cycle can be effectively arrested by 1.0 mmol/L LA. Furthermore, as important ECM proteins, glucose-induced FN and collagen-I expression in MCs results in the accelerated progression of glomerulosclerosis [28]. In our experiments, the secretion of FN and collagen-I was also stimulated by high glucose. Here, we observed that LA modulated the secretion of FN and collagen-I in a dose-dependent manner according to ELISA assays; LA shows significantly facilitative effects on ECM expression with the final concentration 0.25 mM, while inhibiting it with the concentration of 1.0 mM.

It has been demonstrated that when mTOR is activated, mTORC1 can phosphorylate its two direct downstream targets, p70S6K and 4E-BP1, to regulate cell growth, survival, proliferation, apoptosis, and protein synthesis. Multiple factors such as hyperglycemia and decreased adiponectin have been shown to inhibit AMPK activity in diabetic kidneys, where glucose is a central player [29]. Downregulation of AMPK activity and activation of serine/threonine kinase AKT lead to mTOR activation in diabetic kidneys [19]. The activation of mTOR plays a pivotal role in the renal pathologic characteristic of diabetic nephropathy (DN). These observations prompted us to examine the expression of AMPK, AKT, mTOR, 4E-BP1, and p70S6K in MCs treated with LA. It has been reported that LA can dose-dependently upregulate the expression of phosphorylated AMPK in skeletal muscle cells [6]. In the current study, we first detected a concentration-dependent increase of AMPK activity in MCs treated with LA (0.1–1.0 mM). We also observed that AKT in these cells were significantly activated and that 0.25 mM LA led to the highest levels of AKT phosphorylation. It is noteworthy that LA also exhibited a twofold effect on mTOR/p70S6K/4E-BP1 signaling. (1) LA at a final concentration 0.25 mmol/L enhanced the expression of phosphorylated mTOR, p70S6K, and 4E-BP1; this might have been caused by high expression of AKT activation and low expression of AMPK activation. (2) Conversely, LA suppressed their expression at a dose of 1.0 mmol/L, which could have been the result of high expression of AMPK activation and low expression of AKT activation.

Our results demonstrate that treatment with 0.25 mM LA can upregulate AKT and mTOR signaling in high glucose-cultured MCs. It is important to determine whether AKT expression is necessary for mTOR signaling activation induced by 0.25 mM LA. In the current study, the PI3K/AKT inhibitor LY294002 was able to extinguish 0.25 mM LA-induced expression of phosphorylated mTOR, p70S6K, and 4E-BP1. The evidence suggests that AKT is required for mTOR signaling activation caused by 0.25 mM LA. These results are consistent with a previous study by Shen et al. [6] that showed that the CaMKK inhibitor STO-609 could also reverse 1.0 mM LA-induced activation of AMPK in MCs, thereby restoring the mTOR signaling pathway inhibited by 1.0 mM LA, thus suggesting that AMPK is necessary for inhibition of mTOR/p70S6K/4E-BP1 by 1.0 mM LA.

LY294002 also extinguished 0.25 mM LA-induced cell proliferation and ECM secretion. This result suggests that 0.25 mM LA accelerated MC dysfunction by promoting the AKT/mTOR signaling pathway. In addition, STO-609 was able to reverse cell proliferation and ECM secretion inhibited by 1.0 mM LA, suggesting that 1.0 mM LA ameliorated high glucose-induced MC dysfunction by activating the AMPK/mTOR signaling pathway. Previous study showed that, in cultured porcine mesangial cells, LA (50 *μ*mol/L) can prevent upregulation of FN and collagen IV gene expression by inhibiting oxidative stress caused by high glucose [30]. This discrepancy is partly due to the different experimental approaches used in these two studies. It also suggests that in vivo studies including mTOR signaling phosphorylation are needed to further validate these results. Furthermore, changes of oxidative stress should also be detected simultaneously, since the LA can inhibit deposition of mesangial matrix proteins and renal hypertrophy by reducing ROS production. However, our results provide new evidence that upregulation of the AMPK/mTOR signaling plays a protective role in DN [22, 31].

**Figure 9:**
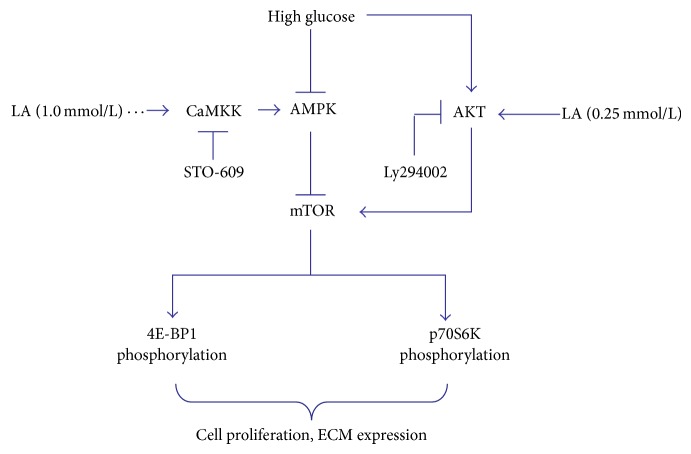
Schematic of the molecular mechanism that LA regulated high glucose-induced mesangial cell proliferation and extracellular matrix secretion.

Taken together, our results demonstrate that LA can regulate high glucose-induced mesangial cell dysfunction by modulating mTOR/4E-BP1/p70S6K signaling, which is mediated by AMPK and AKT activation (Figure 9). These findings provide a novel experimental basis for the possible application of LA in the regulation of diabetes-induced mesangial cell proliferation and matrix expansion in vivo.

## Figures and Tables

**Table 1:** Antibodies, their concentrations and manufacturers in the present studies.

Antibodies	Source	Dilutions	Manufacturer	Catalog number
P-AMPK*α* (Thr172)	Rabbit	1 : 500	Cell signaling	4188S
AMPK*α*	Rabbit	1 : 1000	Cell signaling	4811S
P-AKT (Ser473)	Rabbit	1 : 200	Cell signaling	9271
AKT	Rabbit	1 : 500	Cell signaling	9272
p-mTOR (Ser2448)	Rabbit	1 : 500	Cell signaling	5536S
mTOR	Rabbit	1 : 1000	Cell signaling	2983S
P-p70S6K (Thr389)	Rabbit	1 : 500	Cell signaling	9234S
p70S6K	Rabbit	1 : 1000	Cell signaling	2708S
P-4EBP1 (Thr70)	Rabbit	1 : 500	Cell signaling	9455S
4E-BP1	Rabbit	1 : 1000	Cell signaling	9644S
*β*-Actin	Rabbit	1 : 1000	Santa Cruz	SC-130657

## References

[1] Packham D. K., Alves T. P., Dwyer J. P., Atkins R., De Zeeuw D., Cooper M., Shahinfar S., Lewis J. B., Lambers Heerspink H. J. (2012). Relative incidence of ESRD versus cardiovascular mortality in proteinuric type 2 diabetes and nephropathy: results from the DIAMETRIC (Diabetes Mellitus Treatment for Renal Insufficiency Consortium) database. *The American Journal of Kidney Diseases*.

[2] Reutens A. T., Atkins R. C. (2011). Epidemiology of diabetic nephropathy. *Contributions to Nephrology*.

[3] Conserva F., Pontrelli P., Accetturo M., Gesualdo L. (2013). The pathogenesis of diabetic nephropathy: focus on microRNAs and proteomics. *Journal of Nephrology*.

[4] Steffes M. W., Østerby R., Chavers B., Mauer S. M. (1989). Mesangial expansion as a central mechanism for loss of kidney function in diabetic patients. *Diabetes*.

[5] Melhem M. F., Craven P. A., Liachenko J., DeRubertis F. R. (2002). *α*-lipoic acid attenuates hyperglycemia and prevents glomerular mesangial matrix expansion in diabetes. *Journal of the American Society of Nephrology*.

[6] Shen Q. W., Zhu M. J., Tong J. (2007). Ca2+/calmodulin-dependent protein kinase kinase is involved in AMP-activated protein kinase activation by *α*-lipoic acid in C2C12 myotubes. *The American Journal of Physiology—Cell Physiology*.

[7] Lee W. J., Lee I. K., Kim H. S. (2005). *α*-lipoic acid prevents endothelial dysfunction in obese rats via activation of AMP-activated protein kinase. *Arteriosclerosis, Thrombosis, and Vascular Biology*.

[8] Park K.-G., Min A.-K., Koh E. H., Kim H. S., Kim M.-O., Park H.-S., Kim Y.-D., Yoo T.-S., Jang B. K., Hwang J. S., Kim J. B., Choi H.-S., Park J.-Y., Lee I.-K., Lee K.-U. (2008). Alpha-lipoic acid decreases hepatic lipogenesis through adenosine monophosphate-activated protein kinase (AMPK)-dependent and AMPK-independent pathways. *Hepatology*.

[9] Targonsky E. D., Dai F., Koshkin V., Karaman G. T., Gyulkhandanyan A. V., Zhang Y., Chan C. B., Wheeler M. B. (2006). *α*-Lipoic acid regulates AMP-activated protein kinase and inhibits insulin secretion from beta cells. *Diabetologia*.

[10] Golbidi S., Badran M., Laher I. (2011). Diabetes and alpha lipoic acid. *Frontiers in Pharmacology*.

[11] Saha A. K., Xu X. J., Balon T. W., Brandon A., Kraegen E. W., Ruderman N. B. (2011). Insulin resistance due to nutrient excess: is it a consequence of AMPK downregulation?. *Cell Cycle*.

[12] Diesel B., Kulhanek-Heinze S., Höltje M., Brandt B., Höltje H.-D., Vollmar A. M., Kiemer A. K. (2007). *α*-lipoic acid as a directly binding activator of the insulin receptor: protection from hepatocyte apoptosis. *Biochemistry*.

[13] Lee S. J., Kim S. H., Kang J. G., Kim C. S., Ihm S.-H., Choi M. G., Yoo H. J. (2011). Alpha-lipoic acid inhibits endoplasmic reticulum stress-induced cell death through PI3K/Akt signaling pathway in FRTL5 thyroid cells. *Hormone and Metabolic Research*.

[14] Bitar M. S., Ayed A. K., Abdel-Halim S. M., Isenovic E. R., Al-Mulla F. (2010). Inflammation and apoptosis in aortic tissues of aged type II diabetes: amelioration with *α*-lipoic acid through phosphatidylinositol 3-kinase/Akt- dependent mechanism. *Life Sciences*.

[15] Xie R., Li X., Ling Y. (2012). Alpha-lipoic acid pre- and post-treatments provide protection against *in vitro* ischemia-reperfusion injury in cerebral endothelial cells via Akt/mTOR signaling. *Brain Research*.

[16] Hardie D. G. (2011). Signal transduction: how cells sense energy. *Nature*.

[17] Wiza C., Nascimento E. B. M., Ouwens D. M. (2012). Role of PRAS40 in Akt and mTOR signaling in health and disease. *The American Journal of Physiology—Endocrinology and Metabolism*.

[18] Lieberthal W., Levine J. S. (2012). Mammalian target of rapamycin and the kidney. I. The signaling pathway. *American Journal of Physiology—Renal Physiology*.

[19] Lieberthal W., Levine J. S. (2009). The role of the mammalian target of rapamycin (mTOR) in renal disease. *Journal of the American Society of Nephrology*.

[20] Eid A. A., Ford B. M., Bhandary B., Cavaglieri R. D. C., Block K., Barnes J. L., Gorin Y., Choudhury G. G., Abboud H. E. (2013). Mammalian target of rapamycin regulates nox4-Mediated podocyte depletion in diabetic renal injury. *Diabetes*.

[21] Liu L., Hu X., Cai G. Y., Lv Y., Zhuo L., Gao J.-J., Cui S.-Y., Feng Z., Fu B., Chen X. M. (2012). High glucose-induced hypertrophy of mesangial cells is reversed by connexin43 overexpression via PTEN/Akt/mTOR signaling. *Nephrology Dialysis Transplantation*.

[22] Xu W.-W., Guan M.-P., Zheng Z.-J., Gao F., Zeng Y.-M., Qin Y., Xue Y.-M. (2014). Exendin-4 alleviates high glucose-induced rat mesangial cell dysfunction through the ampk pathway. *Cellular Physiology and Biochemistry*.

[23] Livak K. J., Schmittgen T. D. (2001). Analysis of relative gene expression data using real-time quantitative PCR and the 2-ΔΔCT method. *Methods*.

[24] Wang Y., Li X., Guo Y., Chan L., Guan X. (2010). *α*-Lipoic acid increases energy expenditure by enhancing adenosine monophosphate-activated protein kinase-peroxisome proliferator-activated receptor-*γ* coactivator-1*α* signaling in the skeletal muscle of aged mice. *Metabolism: Clinical and Experimental*.

[25] Yi X., Xu L., Hiller S., Kim H.-S., Nickeleit V., James L. R., Maeda N. (2012). Reduced expression of lipoic acid synthase accelerates diabetic nephropathy. *Journal of the American Society of Nephrology*.

[26] Feng B., Yan X. F., Xue J. L., Xu L., Wang H. (2013). The protective effects of *α*-lipoic acid on kidneys in type 2 diabetic Goto-Kakisaki rats via reducing oxidative stress. *International Journal of Molecular Sciences*.

[27] Gao J., Wang F., Wang W., Su Z., Guo C., Cao S. (2014). Emodin suppresses hyperglycemia-induced proliferation and fibronectin expression in mesangial cells via inhibiting cFLIP. *PLoS ONE*.

[28] Kanwar Y. S., Sun L., Xie P., Liu F.-Y., Chen S. (2011). A glimpse of various pathogenetic mechanisms of diabetic nephropathy. *Annual Review of Pathology: Mechanisms of Disease*.

[29] Hallows K. R., Mount P. F., Pastor-Soler N. M., Power D. A. (2010). Role of the energy sensor AMP-activated protein kinase in renal physiology and disease. *American Journal of Physiology: Renal Physiology*.

[30] Catherwood M. A., Powell L. A., Anderson P., McMaster D., Sharpe P. C., Trimble E. R. (2002). Glucose-induced oxidative stress in mesangial cells. *Kidney International*.

[31] Zhuo L., Fu B., Bai X. (2011). NAD blocks high glucose induced mesangial hypertrophy via activation of the sirtuins-AMPK-mTOR pathway. *Cellular Physiology and Biochemistry*.

